# Building Culturally Centered System Dynamics Logic Models for the Brown Buttabean Motivation Organization: Protocol for a Systems Science Approach

**DOI:** 10.2196/44229

**Published:** 2023-06-16

**Authors:** Faasisila Savila, Truely Harding, Boyd Swinburn, Warwick Bagg, Dave Letele, Fuatino Laban, Felicity Goodyear-Smith

**Affiliations:** 1 School of Population Health University of Auckland Auckland New Zealand; 2 School of Medicine University of Auckland Auckland New Zealand; 3 Brown Buttabean Motivation Manukau Auckland New Zealand

**Keywords:** systems logic model, cognitive mapping interview, group model building, community network, healthy lifestyle, weight reduction programs

## Abstract

**Background:**

Brown Buttabean Motivation (BBM) is an organization providing support for Pacific people and Indigenous Māori to manage their weight, mainly through community-based exercise sessions and social support. It was started by DL, a man of Samoan and Māori descent, following his personal weight loss journey from a peak weight of 210 kg to less than half that amount. DL is a charismatic leader with a high media profile who is successful in soliciting donations from corporations in money and kindness. Over time, BBM’s activities have evolved to include healthy eating, food parcel provision, and other components of healthy living. A co-design team of university researchers and BBM staff are evaluating various components of the program and organization.

**Objective:**

The purpose of this study is to build culturally centered system dynamics logic models to serve as the agreed theories of change for BBM and provide a basis for its ongoing effectiveness, sustainability, and continuous quality improvements.

**Methods:**

A systems science approach will clarify the purpose of BBM and identify the systemic processes needed to effectively and sustainably achieve the study’s purpose. Cognitive mapping interviews with key stakeholders will produce maps of their conceptions of BBM’s goals and related cause-and-effect processes. The themes arising from the analysis of these maps will provide the initial indicators of change to inform the questions for 2 series of group model building workshops. In these workshops, 2 groups (BBM staff and BBM members) will build qualitative systems models (casual loop diagrams), identifying feedback loops in the structures and processes of the BBM system that will enhance the program’s effectiveness, sustainability, and quality improvement. The Pacific and Māori team members will ensure that workshop content, processes, and outputs are grounded in cultural approaches appropriate for the BBM community, with several Pacific and Māori frameworks informing the methods. These include the Samoan *fa’afaletui* research framework, which requires different perspectives to be woven together to create new knowledge, and *kaupapa Māori*–aligned research approaches, which create a culturally safe space to conduct research by, with, and for Māori. The Pacific *fonofale* and Māori *te whare tapa whā* holistic frameworks for interpreting people’s dimensions of health and well-being will also inform this study.

**Results:**

Systems logic models will inform BBM’s future developments as a sustainable organization and support its growth and development beyond its high dependence on DL’s charismatic leadership.

**Conclusions:**

This study will adopt a novel and innovative approach to co-designing culturally centered system dynamics logic models for BBM by using systems science methods embedded within Pacific and Māori worldviews and weaving together a number of frameworks and methodologies. These will form the theories of change to enhance BBM’s effectiveness, sustainability, and continuous improvement.

**Trial Registration:**

Australian New Zealand Clinical Trial Registry ACTRN 12621-00093-1875; https://www.anzctr.org.au/Trial/Registration/TrialReview.aspx?id=382320

**International Registered Report Identifier (IRRID):**

PRR1-10.2196/44229

## Introduction

### Background to Brown Buttabean Motivation

In 2014, DL, a man of Samoan and Māori descent, set up an organization called Brown Buttabean Motivation (BBM) in south Auckland, Aotearoa New Zealand. DL had a personal weight loss journey from a peak weight of 210 kg to less than half that amount [1]. BBM provides support for Pacific people and Indigenous Māori to manage their weight, mainly through community-based exercise sessions and social support. Pacific and Māori people have much higher rates of obesity- and weight-related diseases than non-Pacific and non-Māori people.

While exercise boot camps were the entry point for community engagement, over time BBM’s activities have evolved to include healthy eating and other components of healthy living, which are woven into a collective journey toward health and well-being [2]. DL is the high-profile leader of BBM, which is now a limited liability company. BBM’s operations staff includes both paid employees and volunteers. Its funding comes from a variety of sources, including philanthropy, the Ministry of Health, and donations through its Just Move Charitable Health Trust funding arm. Its members, largely but not exclusively of Māori and Pacific ethnicities, are now located not only in south Auckland but also in the west of the city and other parts of Aotearoa New Zealand.

Researchers at the University of Auckland were asked by BBM to co-design an evaluation of the various components of the program and the organization. A qualitative study has been conducted to understand how participants experience and engage with BBM as a grassroots, Pacific-, and Māori-led holistic health program [2]. Most interventions to address obesity do not result in sustained weight loss over time, and a review of the literature recommended “Māori and Pacific-led, culturally tailored weight loss programs that promote holistic, small, and sustainable lifestyle changes delivered in socially appropriate contexts” [3]. A pre-post longitudinal quantitative cohort study is underway to evaluate the effectiveness of BBM for sustained health and well-being outcomes, especially weight loss [4]. A further study is underway to conduct a mixed methods process evaluation of BBM’s community engagement through in-person, social, and news media outreach activities with respect to the health and well-being of the Māori and Pacific communities over time [5].

### Systems Science

#### Overview

A system is an interconnected set of elements coherently organized in a way that achieves something [6]. The structure of a system is therefore comprised of a complex interplay of 3 types of things: elements, connections, and a function or purpose. The meanings that people attribute to these elements may or may not be universally shared. Understanding these components is required to accurately model the system. People may have different mental models of the same events and data, and visual mapping and modeling tools can help reveal these different interpretations and combine them into an overarching perspective. Once the structure is identified, it can be altered in order to make changes to the system.

#### Logic Models of Change

A logic model is a theory of change. It demonstrates the inputs (what goes in), outputs (what comes out), outcomes (what results from this), and the relationships between them. Often, logic models are linear in nature (eg, inputs leading to processes, leading to outputs, leading to immediate impacts, and leading to long-term outcomes), showing the intentions, assumptions, and rationale of an intervention and how it can influence a system to achieve an outcome. A dynamic model identifies and uses feedback loops and can show what things may lead to another and different factors that might lead to change, both those that can be controlled and others unrelated to the program and hence out of the organization’s control [6]. A systems approach that can incorporate feedback loops will create sustainability in a rapidly evolving, responsive program.

Logic models are graphic depictions of the relationships between resources, activities, outcomes, and impacts of a program or a system. They have been used in a variety of settings, for example, to inform the development of interventions such as a rehabilitation service for youth with concussion [7,8], mobile health support for physical activity in people with spinal cord injuries [9], and digital self-management in inflammatory bowel disease [10]. They have been used to understand how digital health interventions for self-management might be used to facilitate effective behavior change [11], how to design a plan to transform a health care system [12], and to capture complexities in systematic and scoping reviews [13]. Tools to create dynamic logic models include cognitive mapping interview (CMI), sometimes referred to as causal mapping and group model building (GMB). To appropriately represent the theory of change within a complex adaptive system, like BBM, system dynamics logic models need to account for the feedback loops intrinsic to the system.

#### Cognitive Mapping Interviewing

CMI is a visual technique that captures a stakeholder’s perspective on a problem or issue and how they or their organization relate to the issue. This tool is used in systems research to view issues (for example, low fruit and vegetable consumption in children [14]) as emerging from complex and adaptive systems. A set process is followed during a semistructured interview to order and reflect the interviewee’s view of the social system. The process of creating the CMI map aids participants’ reflection and sense-making. The map produced during each interview consists of a network of nodes and arrows as links that are usually linear in structure. The themes that emerge from the analysis of the individual maps can then be combined to form a composite map, presenting the subjective data in a diagrammatic form that gives a holistic and systemic view of an issue. CMI has most often been applied to complex management problems and operational or action research. CMI was used in a health services project to develop a theoretical framework for implementing innovative practices in primary health care management in Aotearoa New Zealand [15], and other studies internationally have used similar system dynamics methods [16].

#### Group Model Building

GMB is a qualitative method that aims to engage stakeholders to collectively consider the dynamics of complex problems [17]. It involves a facilitation or modeling team and a group of stakeholders who cobuild a model to solve specific problems within a complex system [18,19]. GMB, as a participatory method, extracts the knowledge and understandings of a problem from people who engage with it by using a series of facilitated, structured discussions. GMB has applications ranging from problem definition to simulation system dynamics modeling [20]. In other words, in GMB sessions, people provide their individual perspectives on the problem, its structure, the resulting dynamic model, as well as its impacts and solutions. These views are improved by knowledge sharing among people, discussions, and group analysis. When a consensus model is achieved, the group then identifies leverage points within the system and the actions needed to reorient the system to resolve the problem. This approach ensures that people’s perspectives and understandings are included, thereby increasing their commitment to execute their action tasks [21]. The GMB process enables the creation of visual system maps that identify the feedback loops that are holding a problem in place or that can be reoriented to reduce the problem.

### Cultural Approaches and Frameworks

#### Overview

There are several Māori and Pacific frameworks and models that are appropriate to use in Aotearoa New Zealand to inform systems science approaches. The core personal and social values of Māori and Pacific people are love, humility, respect, and relationship. Connectedness and collectivity are key.

#### Kaupapa Māori

*kaupapa Māori* aligned research ensures that the experiences and outcomes of research are positive for Māori communities [22]. kaupapa Māori research prioritizes Māori knowledge, language, and customs research practice [23]. There are 5 key principles: tino rangatiratanga (self-determination), taonga tuku iho (cultural aspirations), ako Māori (Māori worldview), whānau (family), and kia piki ake i nga raruraru o te kainga (socioeconomic mediation) [24,25]. These key principles will guide the CMIs and GMB workshops to ensure that the researchers and the research process provide a culturally safe environment for our participants and that the outcomes of the study have a positive impact on Māori communities [25,26].

#### Fa’afaletui

The Samoan *fa’afaletui* research framework also informs our study design. *Fa’afaletui* requires different perspectives woven together to create new knowledge (from “ways of” [*fa’a*] “weaving together” [*tui*] deliberations of different groups or “houses”[*fale*]) and is derived from the Pacific philosophy of connectedness and a collective holistic approach [27,28]. This fits well with the use of a mixed methods approach with a range of different types and sources of data. This research framework is also reflected in our team, which includes researchers with expertise in obesity reduction, co-design, and systems science, as well as the Māori and Pacific investigators outlined below. In line with the *fa’afaletui* framework, the collective approach of our study places greater value on the research because of the shared vision and support of all members (researchers, BBM workers, and community members), which intertwines the skills and perspectives within the research team to work toward a shared goal [26]. The bodies of knowledge that each person brings are woven together to conduct this *mahi* (work). Different systems science methodologies will also be interwoven to create our system model of change. 

#### Fonofale and Te Whare Tapa Whā

*Fonofale* (Pacific) [29] and *te whare tapa whā* (Māori) [30] models of inquiry are holistic frameworks for interpreting people’s dimensions of health and well-being. These frameworks use the metaphor of a Samoan or Māori meetinghouse. In the fonofale model, the floor is the extended family and community; the 4 house pillars, or pou, represent physical, mental, spiritual, and sociodemographic components of health and well-being; and the cultural practices, beliefs, and values that shelter people are the roof. The fonofale is surrounded by the physical environment and the sociopolitical context and is set at a point in time. Similarly, the Māori te whare tapa whā model has the foundation of the house (whare) as the land and place of belonging (taha whenua), and the 4 posts are physical (taha tinana), mental (taha hinengaro), spiritual (taha wairua), and family (taha whānau) well-being. Good health requires that these 4 posts be balanced and in harmony with each other [31].

### Why BBM May Have System Issues That It Would be Helpful to Address

BBM has a core vision to provide, in DL’s own words, “a health hand-up” for Māori and Pacific people. This is a broad vision that, at its core, has the goal of improving the health of *whānau* (family). Consequently, BBM engages with the community to enhance physical, spiritual, mental, and social health. Specific examples of activities include addressing food insecurity, providing a hub for exercise, encouraging vaccination, and meeting the needs of those displaced by recent flooding in Auckland and Hawkes Bay. BBM changes its focus in direct response to perceived community needs. There is a risk that BBM will overextend and not be sustainable. The development of a dynamic logic model of change can help identify organizational aspects that are functioning well independently and those that rely on DL’s input. This knowledge may help BBM adapt and remain an effective and sustainable entity in his absence.

### Aim

We aim to build a culturally centered system dynamics logic model to serve as the agreed theories of change for BBM and provide a basis for its ongoing effectiveness, sustainability, and continuous quality improvements. To achieve this, we are combining systems science approaches with several Pacific and Māori frameworks for health and processes for research.

## Methods

### Overview

This research will be *kaupapa Māori*–aligned. The principal investigator is of Samoan and Māori descent; our team also includes a Māori researcher to guide the team on *kaupapa Māori* research methodologies; and BBM staff, including DL, who identifies as Samoan and Māori, are coresearchers. The principal investigator and Māori researcher have been involved in all aspects of study conceptualization, design, and processes and will oversee data analysis and interpretation to ensure that the research is mana-honoring and safe for Māori. In alignment with *kaupapa Māori*, this research has been co-designed with BBM staff to ensure that the needs of the organization and the community are of the utmost importance. This is an example of the key principle of *whānau* . In this research, *whānau* refers both to people in a family (immediate and extended) and also to how Māori practice *whanaungatanga* (the way family members interact with each other) [25,30]. Everyone involved in the study is part of the research *whānau* , and the knowledge that each person brings to this research *whānau* is shared and valued.

The Pacific (*fonofale*) [28] and Māori (*te whare tapa whā*) [29] holistic frameworks for health will inform our research. Although its initial focus was on weight loss, particularly for the Pacific and Māori communities, BBM has evolved to provide social support for the overall health and well-being of its community, in line with the *fonofale* and *te whare tapa whā* frameworks.

There are 3 phases to our research. In phase 1, key stakeholders will be interviewed to produce cognitive maps of BBM’s goals and cause-and-effect processes.

These maps will inform the main themes for exploration and questions in phase 2, which involves 2 series of GMB workshops, one with BBM staff and another with BBM members. During these workshops, participants will build visual system models. The process will identify feedback loops between the elements, structures, and processes of the BBM system, providing the theories of change for BBM to drive its effectiveness, sustainability, and continuous quality improvement. The goal of the staff workshop is to map the dynamics of the key parts of the BBM program itself that relate to its quality and sustainability, such as the continuous development of staff, program content, and funding. The members’ workshop will focus on how to maintain and enhance participant engagement with BBM because program engagement is the best determinant of successful long-term weight loss. In the final phase, these visual maps will be used to create an integrated system dynamics model of change and the associated action plans needed to reorient BBM systems so that they can deliver optimum benefits for the BBM community.

### Phase 1: Cognitive Mapping Interviews

#### Overview

The CMIs for this research will be used to identify a shared understanding of the purposes and goals of BBM. They will also identify the components and cause-and-effect processes of success and sustainability for BBM to deliver its purposes and goals. These concepts will initially become the indicators of change to inform the questions for further exploration in the GMB workshops.

#### CMI Recruitment

Between 5 and 10 key informants will be recruited as CMI participants. Participants will include 2 to 3 key people from the following groups: BBM staff members, long-term community members (including volunteers) who are focused on sustained engagement with BBM, and relevant funding representatives to BBM who are interested in the program meeting its purpose and remaining sustainable. Inclusion criteria for BBM staff are the founder and chief of operations; for male and female participants, they are adult (≥18 years) community members who have engaged with BBM for at least three years; and for funding representatives, the criteria are known funders of BBM projects or have an interest in potentially funding BBM projects. Participants will all give informed consent. Given the small number of participants required for the study’s recruitment, they will be individually approached by our lead researcher, who has strong connections within the BBM community.

While standard qualitative approaches using thematic analysis require continuing recruitment until data saturation is reached, in this study the data are the complex maps created during the interviews. The purpose of the CMIs is not to get definitive maps but to better understand how to frame and define the central “problem” being placed on the table as the purpose of the GMB workshops.

The interviews will generally be 30 to 60 minutes long with a researcher hand-drawing the maps. Seeking clarification that the relationships and directions of influence are being accurately represented will help keep the interviewees engaged.

#### Developing the CMI Questionnaire

An open-ended questionnaire will be co-designed in consultation with all of the investigators and our BBM partners. This will ensure that the questions are inclusive of the frameworks and approaches guiding this study. For instance, prompts will support participants to think holistically, upholding Pacific and Māori cultural values housed within the respective models described above. The questionnaire will be piloted prior to the CMIs and assessed for cultural relevance. We will use the “purpose of BBM” for the lead staff and the funders to identify common understandings of the BBM purpose and what drivers help if fulfill that purpose and what the consequences would be from achieving that purpose. For the participants, the central issue will be engagement with BBM—the antecedents that drive participation and the consequences of high participation.

#### CMI Processes

The purpose of the CMIs is to seek the views of these key stakeholders as to the purpose of BBM, what would ensure its success and sustainability in achieving that purpose, and what the outcomes for the community would be if BBM achieved that purpose. The interviews will be conducted by the lead researcher (FS), who is of Māori and Samoan descent, and an associate researcher (TH), who is of Māori descent. The mapping will be conducted by BS, who is experienced in CMI research. FS and TL have undergone training in CMI and GMB from Synergia, an analytical company with considerable experience and expertise in designing and developing health networks. Their combined knowledge and expertise will ensure appropriate cultural formalities to allow participants to feel comfortable and culturally safe during the interview process. Interviews can be conducted in English, Samoan, or Te Reo (Māori) if desired by the participants. To align with *kaupapa Māori* methodology, the interview will begin with the lead researcher offering a *karakia* (prayer) to appropriately open and guide the interview process. A closing prayer will be shared to bring the interview to an end for everyone involved. Interviews will be audiotaped to allow the researchers to refer back to their maps for review.

Consistent with Māori and Pacific preferences for engagement, we prefer that interviews occur face-to-face either at a BBM gym (for BBM staff) or at a location suitable to the participant. For external collaborators (eg, funding representatives), the researchers will offer a university campus meeting room (south or central Auckland), or participants may choose their place of employment. If face-to-face is not suitable, a virtual meeting platform (eg, Zoom) will be used, in which case only sound will be recorded. During the interview, one researcher will lead the interview guided by the questionnaire, and the other will construct a hand-drawn cognitive map. The interviewee will review the interviewer’s interpretation of comments, illustrated in the map, throughout the session. The interview transcript, map, and consent form will be linked by participant number rather than name to maintain confidentiality. Participants will be given a small *koha* (gift) at the conclusion of the interview to thank them for their involvement and acknowledge their contribution to the study. Following the interview, the hand-drawn maps will be refined and drawn digitally. Participants will be emailed the transcript and the digital version of the cognitive map constructed during their interview, and they will be called to verify them and take any suggestions they offer.

The results from the interviews will form the basis of the GMB workshops. The purpose of using CMI is to generate an informed base of knowledge about what team members, community members, and funders believe are the strengths and challenges of delivering a holistic, effective, and sustained service for the community. In particular, the themes that emerge will provide the key indicators for the expected changes over time. These will form the “reference behaviors” for the GMB workshops. A reference behavior is a key indicator within the system that has a changing pattern over time and, looking into the future, has a more or less desirable trajectory. For example, participant engagement with the BBM sessions typically starts high with enrollment but then wanes over time. The less desired trajectory is a greater disengagement profile, in the future and the more desired trajectory is greater engagement because this is known to be a strong predictor of long-term weight loss maintenance. For sustainability, increasing funding will be needed over time, with declines in funding being the less desirable trajectory.

### Phase 2: Group Model Building

GMB problems can be defined in terms of solutions; for example, how BBM may increase the degree and length of engagement of participants with BBM.

#### Developing the GMB Scripts

The structured scripts to use in the GMB workshops will be co-designed with the BBM coresearchers involved in this research project to align the research processes with the community’s needs. Scriptapedia is a repository of short, facilitated activities (scripts). It is an evolving, open-source handbook of contributed scripts that can be considered best practices, promising, or under development [31]. These scripts can be easily edited to suit the local environment, cultures, and the problem or issue being investigated.

We will weave relevant scripts together to create the facilitated, structured discussions for our GMB workshops. This will help to ensure that the workshops achieve the desired outcomes of creating systems maps and action priorities, as well as upskill the participants in their systems thinking so that they can better understand the nature of the issue being mapped and the ways to orient the BBM systems for improved outcomes. The interactions within a group during a GMB workshop need to make the best use of their time and ensure that the overall process moves forward in an organized fashion, ultimately providing insights for the research team and stakeholders [20].

#### GMB Workshop Recruitment

Participants will be recruited for 2 series of workshops, with 15-20 participants per series. One series will be with BBM employees and volunteers (staff), and the other with active BBM community members, including volunteers, who engage with the organization on a regular basis (members). Each series will consist of 3 workshops with about 2 weeks between them. All workshops will be held at one of the BBM gyms in either south or west Auckland or the University of Auckland’s south Auckland campus. Recruitment for both series will be a collaborative process between researchers and BBM staff to negotiate recruitment at a time and place suitable to participants and BBM. For BBM community members to be invited, they will need to have been active members for at least 6 months prior to the planned workshops and plan to continue to engage on a regular basis. BBM holds attendance records and member details, so the research team will work with BBM staff to purposively invite a representative balance of male and female adult (aged ≥18 years) members to participate. There will be no ethnic exclusions, but the aim is to have predominantly Pacific and Māori participants. People who have previously participated in the CMIs and those who are not fluent in English, Māori, or Samoan will be excluded. All participants will give informed consent and will be offered *koha* (reimbursement) for their time, in line with our ethics approval.

#### GMB Processes

The GMB workshops will be co-designed by the research team and the coresearchers from BBM (DL and FL) to ensure that the questions asked, processes put in place, and language used are appropriate for the participants. Prior to the first series of workshops, the researchers will carry out training with a single series of mock workshops with a small number of Māori and Pacific staff and students as test participants to trial the structured scripts and activities for flow, cultural appropriateness, and timing.

Each workshop will follow a defined structure and process. In alignment with *kaupapa Māori*, a *karakia* (prayer) will be used to open and close the workshops. *Kai* (food) and *koha* (a small gift) for participants will be included. *Kai* is a sign of hospitality, an example of showing *manaakitanga* (kindness) and willingness to create an inviting environment. *Koha* is a sign of reciprocity. The participants will be sharing their knowledge and perspectives; therefore, we must acknowledge their valuable contribution and the time they have taken to take part in our research project.

The aim is to keep each workshop to less than 3 hours and to allow for appropriate *whakawhanaungatanga* (introductions and group activities) and breaks, as well as covering the necessary content.

Previous co-design research combining Indigenous and Pacific knowledge and systems thinking will help shape our processes. The goal of workshop 1 will be to orientate the groups and develop a shared understanding of the key issues through a series of constructed activities through the scripts, designed and edited from Scriptapedia. The first will be “hopes and fears” [32], which will assist with the *whakawhanaungatanga* (getting to know each other) process. This will be followed by another script called “reference behaviors over time” to help participants start thinking dynamically by looking at the key indicators (reference behaviors) identified from the CMIs. This is followed by a third script, “connection circles,” using software called STICKE (systems thinking in community knowledge exchange), which has been developed in collaboration with Victorian communities through research undertaken by Deakin University. The process helps participants start thinking about the components of an issue and their relationships, or cause and effect (eg, increasing stories and photos through the BBM Facebook page and media increases the sense of engagement with BBM among participants).

In workshop 2, participants will start to construct a visual map of the BBM organization through a series of “feedback” loops, also known as causal loop diagrams (CLDs), which demonstrate sustainable cause and effect loops; a classic example is the chicken and egg loop. The CLDs are also developed in STICKE and are a continuation of the connection circles activity.

In the third workshop, the participants will review and explore further these CLDs to ensure they fully represent their perspectives and visual maps, make any further additions or adjustments, and identify future actions using the identified system levers. Action ideas would be assessed based on practicality, whether they are achievable, and the size of the effect.

Quality criteria for phase 2 are the use of *kaupapa Māori* principles to ensure that participants feel culturally safe and are treated with respect; the researcher training sessions of mock workshops; the use of standardized scripts; support in the development of CLDs; and a final reflective session to make additions and adjustments and make sure their action ideas are practical, feasible, and achievable. Additionally, *kaupapa Māori* allows for flexibility; while each workshop has a defined structure and process, researchers are willing to adjust those processes if they are not helpful for the participants or make anyone feel uncomfortable. Finally, the lead facilitators of these workshops will remove any jargon or intimidating terms that may discourage participants from engaging in the workshops. It is important to note that it is the researcher’s responsibility to ensure that the way the content is communicated is just as important as the content itself. The lead Māori and Pacific researchers will be constantly checking in with participants to encourage feedback, increase engagement, and establish trust with them.

### Phase 3: The System Dynamics Logic Model and Action Plan

In phase 3, the outputs of the CMI will inform the development of 2 system dynamic logic models, one from BBM staff and one from BBM members. In alignment with our methodologies for this study, creating a dynamic model allows for more community input and engagement. Māori and Pacific communities are unique population groups that require a system that is tailored to their sociocultural values and beliefs.

By focusing on creating systems models with feedback loops rather than linear models, this study aims to incorporate and highlight the actions and factors that specifically relate to Māori and Pacific communities. This is done by the research team and members of the BBM team using the visual systems maps created during the GMB workshops to build a system model of change that benefits the BBM community. BBM senior staff members, including DL, and the research team will collectively develop an action plan based on the learnings from these models.

Quality criteria for phase 3 include the final 2 system dynamics logic models, one from BBM staff and one from BBM members, informed by the robust processes conducted in phases 1 and 2. Adherence to Māori and Pacific cultural values and principles means that the models and subsequent action plans incorporate and highlight the actions and factors that specifically relate to these communities.

### Ethics Approval

Ethical approval for the evaluation study was given by the Health and Disability Ethics Committee New Zealand for 3 years on June 30, 2021 (21/STH/122), with an amendment for the cognitive mapping component of the project approved on November 9, 2022.

Participants in the CMI and GMB will be provided with participant information sheets and will give informed consent. All participants will be anonymous. Participants will receive NZD $50 (US $31) supermarket vouchers per session as compensation for their time.

## Results

The 2 logic models will inform BBM’s future developments as a sustainable organization. The end result of this study will be an action plan based on the logic model and system levers for how BBM can optimize its effectiveness, sustainability, and quality improvement.

BBM was developed by DL, who is a charismatic leader and continues to actively drive the organization in new directions to meet arising community needs. The action plan will include consideration of how and whether BBM can continue to grow and expand independently of DL’s charismatic leadership.

## Discussion

### Summary of Findings

This study aims to build culturally centered system dynamics logic models to serve as the agreed theories of change for BBM and provide a basis for its ongoing effectiveness, sustainability, and continuous quality improvements. To achieve this, we will adopt a novel and innovative approach, using systems science along with collaborative co-design embedded within Pacific and Māori world views and weaving together a number of frameworks and methodologies to create a system dynamics logic model for BBM. These are highly compatible approaches with strong potential synergies.

The final result will be an action plan for BBM to hone the underlying system features identified in the 2 logic models.

A logic model that takes the dynamics of the system into account with feedback loops that improve effectiveness, sustainability, and quality can be explicitly considered in action plans for the ongoing success of BBM. The use of STICKE makes the process of creating the systems maps more accessible and available in real time.

The culturally centered design is to ensure relevance and connection to the cultures and world views of the participants, the BBM team, and their communities, and the innovations and learnings that could come from joining up these approaches will guide the further development of BBM.

### How These Findings May Relate to the Literature

Having developed the models and the plan, we will determine how this compares with other published studies, and how it fits into the existing literature with respect to its interpretations and implications.

### Limitations

The maps developed in the CMI phase will reflect the perceptions of the organizational structure of the key people selected to interview. There is a subjective component to generating these data. While 2 to 3 people selected for each of the 3 categories (staff, members, and funders) will be those seen as key personnel, it is possible that if a larger sample was used, there might be more variability in the information used to inform the GMB. However, we are not aiming to develop definitive themes from the CMIs. Instead, CLDs from these will provide a guiding example for the GMB participants who will go on to identify other major causal drivers to be considered thoroughly by the wider group.

Similarly, an increased number of participants in the GMI workshops might result in more divergency in the created maps.

### Conclusions

This study will adopt a novel and innovative approach to co-designing culturally centered system dynamics logic models for BBM by using systems science methods embedded within Pacific and Māori world views and weaving together a number of frameworks and methodologies. These will form the theories of change to enhance BBM’s effectiveness, sustainability, and continuous improvement.

Programs aiming to change people’s health behaviors and reduce health inequities can be complex and fail to deliver because they do not overcome the barriers to the system and individual change that they encounter. This includes the application of Western principles to ethnic minorities. Beyond the goal of informing BBM on how it may introduce quality improvements and address identified system issues, we hope that our study will provide a framework for other organizations and networks to assess system factors that might be beneficial to address in a culturally centered and safe manner.

## Abbreviations

BBM: Brown Buttabean Motivation
CLD: closed loop diagram
CMI: cognitive mapping interview
GMB: group model building
STICKE: systems thinking in community knowledge exchange

## Data Availability

Data sharing is not applicable to this article as no data sets per se were generated during this study. The data generated during the study consisted of field notes and visual maps hand-drawn and then computerized from the interviews and group map-building workshops.
